# 157 Qualitative Analysis of Infection Preventionists’ Experiences Implementing Enhanced Barrier Precautions in Long-Term Care Facilities

**DOI:** 10.1017/ash.2026.10558

**Published:** 2026-06-23

**Authors:** Lindsay Bow, Akaash Patel, Chiquacta Hodges, Chloe Lewis, Carley Freiji, Valeria Fabre

**Affiliations:** 1 The Johns Hopkins Hospital; 2 John Hopkins Hospital; 3 Johns Hopkins Hospital; 4 Johns Hopkins Health System; 5 Johns Hopkins University School of Medicine

## Abstract

**Background:** Patients undergoing hepato-pancreato-biliary (HPB) surgery routinely need central venous access for chemotherapy or parenteral nutrition. Common postoperative complications of HPB surgeries include cholangitis and transient bacteremia from biliary tract manipulation. Previous studies have suggested that the National Healthcare Safety Network (NHSN) criteria that designate bacteremia secondary to infections such as cholangitis, may be too strict to account for these situations, resulting in central line associated bloodstream infection (CLABSI) misattribution. **Methods:** This retrospective study evaluated bacteremia events within 30 days of an HPB surgical procedure among patients ≥ 18 years old at a large academic medical center in the United States. The study period included January 1, 2024 through October 31, 2025. HPB procedures were identified using operative codes mapped to HPB surgeries by NHSN with bacteremia cases identified electronically. CLABSI cases, as established by NHSN criteria, underwent manual chart review to understand the clinical context. **Results:** There were 1,143 HPB surgeries within the study period. Bacteremia was detected in 35 of them (3.1%). Five of these were ruled in as CLABSI. CLABSIs occurred at an average of 9.6 days postoperatively and 7.6 days after central line placement or access. CLABSIs were due to Enterococcus faecalis, Candida krusei, Prevotella species, Klebsiella oxytoca, and Staphylococcus aureus. Three of the CLABSIs had clinical evidence of cholangitis, but imaging findings were insufficient to meet NHSN criteria for an intraabdominal infection. These cases included transient bacteremia within 24 hours of biliary manipulation (drain placement for a portobiliary fistula, n=1, and drain capping, n=2). The other two CLABSI cases did not undergo biliary manipulation during their hospitalization and had no clear evidence of secondary infection. **Conclusion:** A substantial number of CLABSIs in HPB surgical patients may represent misattribution. Consideration to the unique infection risks of HPB surgical patients such as transient bacteremia related to drain placement and exchange could minimize CLABSI misattribution in this patient population. Ellsworth, M. G., Ausborn, V., Patel, B., Chang, P., & Ostrosky-Zeichner, L. (2023). 2419. Increasing Misattribution of Bacteremia as CLABSIs by NHSN Definitions: A Tertiary Care Center Perspective. Open Forum Infectious Diseases, 10(Suppl 2), ofad500.2039. https://doi.org/10.1093/ofid/ofad500.2039

